# Renal scattered tubular-like cells confer protective effects in the stenotic murine kidney mediated by release of extracellular vesicles

**DOI:** 10.1038/s41598-018-19750-y

**Published:** 2018-01-19

**Authors:** Xiangyu Zou, Soon Hyo Kwon, Kai Jiang, Christopher M. Ferguson, Amrutesh S. Puranik, Xiangyang Zhu, Lilach O. Lerman

**Affiliations:** 1 Division of Nephrology and Hypertension, Mayo Clinic, Rochester, MN USA; 2 Department of Urology, Xinhua Hospital, Shanghai Jiao Tong University School of Medicine, Shanghai, China; 3 Division of Nephrology, Soonchunhyang University Seoul Hospital, Seoul, Korea

## Abstract

To test the hypothesis that intrinsic renal scattered tubular cells (STC-like cells) contribute to repairing injured tubular epithelial cells (TEC) by releasing extracellular vesicle (EV). EV released from primary cultured pig STC-like cells were confirmed by electron microscopy. Antimycin-A (AMA)-induced injured proximal TEC (PK1 cells) were co-cultured with STC-like cells, STC-like cells-derived EV, or EV-free conditioned-medium for 3 days. Cellular injury, oxidative stress and mitochondrial function were assessed. Transfer of mitochondria from STC-like cells to TEC was assessed using Mito-trackers, and their viability by mitochondrial membrane potential assays. STC-like cells-derived EV were intra-arterially injected into mice 2 weeks after induction of unilateral renal artery stenosis. Two weeks later, renal hemodynamics were studied using magnetic-resonance-imaging, and renal fibrosis assessed *ex-vivo*. Cultured STC-like cells released EV that were uptaken by TEC. A protective effect conferred by STC-like cells in AMA-induced TEC injury was partly mimicked by their EV. Furthermore, STC-like cells-EV carried and transferred mitochondrial material to injured TEC, which partly restored mitochondrial function. *In vivo*, STC-like cells-derived EV engrafted in the stenotic kidney, and improved its perfusion and oxygenation. STC-like cells-EV exert protective effects on injured tubular cells *in vitro* and *in vivo*, partly by transferring STC-like cells mitochondria, which remain at least partly functional in recipient TEC.

## Introduction

Ischemic injury is a major cause of kidney injury in many pathophysiological conditions, including renal artery stenosis (RAS)^1^. Renal ischemia can induce injury to renal tubular epithelial cells (TEC), potentially leading to cellular apoptosis or necrosis, and in turn loss of renal function^2^. Nevertheless, TEC have some capacity for repair, following kidney injury. Previous studies showed that a group of renal resident scattered tubular cell (STC) undergo proliferation after injury and contribute to the renal recovery^3,4^. The source of these cells after kidney injury remains unclear, but recent studies suggest that some TEC undergo dedifferentiation to yield STC as main resource for tubular regeneration after injury^5–7^.

Regeneration of TEC after kidney injury may involve paracrine, autocrine, or endocrine actions of reparative cells. Release of membrane extracellular vesicles (EV) is considered as an important pathway of intercellular communication, as EV deliver proteins and genetic materials from donor to target cells. Recent studies have shown that some EV may also contain and deliver mitochondrial materials that can enhance bioenergetics in recipient cells^8–10^. For example, mitochondria from astrocytes can be packaged into large membrane vesicles, and their transfer to neurons alleviates cell injury after stroke^11,12^. Mitochondria are the main energy producing organelles, and their dysfunction may lead to accumulation of reactive oxygen species from the electron transport chain (ETC), release of apoptotic factors, and ultimately cell injury and death^13^. Thus, therapeutic strategies aimed at restoring mitochondrial function might attenuate renal injury^14^. However, whether STC employ this mechanism of intercellular communication in the reparative program of injured TEC remains unknown.

We hypothesized that STC-like cells release EV that are uptaken and deliver mitochondrial materials to injured TEC to initiate a repair program and blunt ischemic renal injury. To this end, we studied the effects of STC-like cells-derived EV on injured TEC both *in vitro* and *in vivo*.

## Results

### Characterization of STC-like cells and STC-like cells-EV

**Figure 1:**
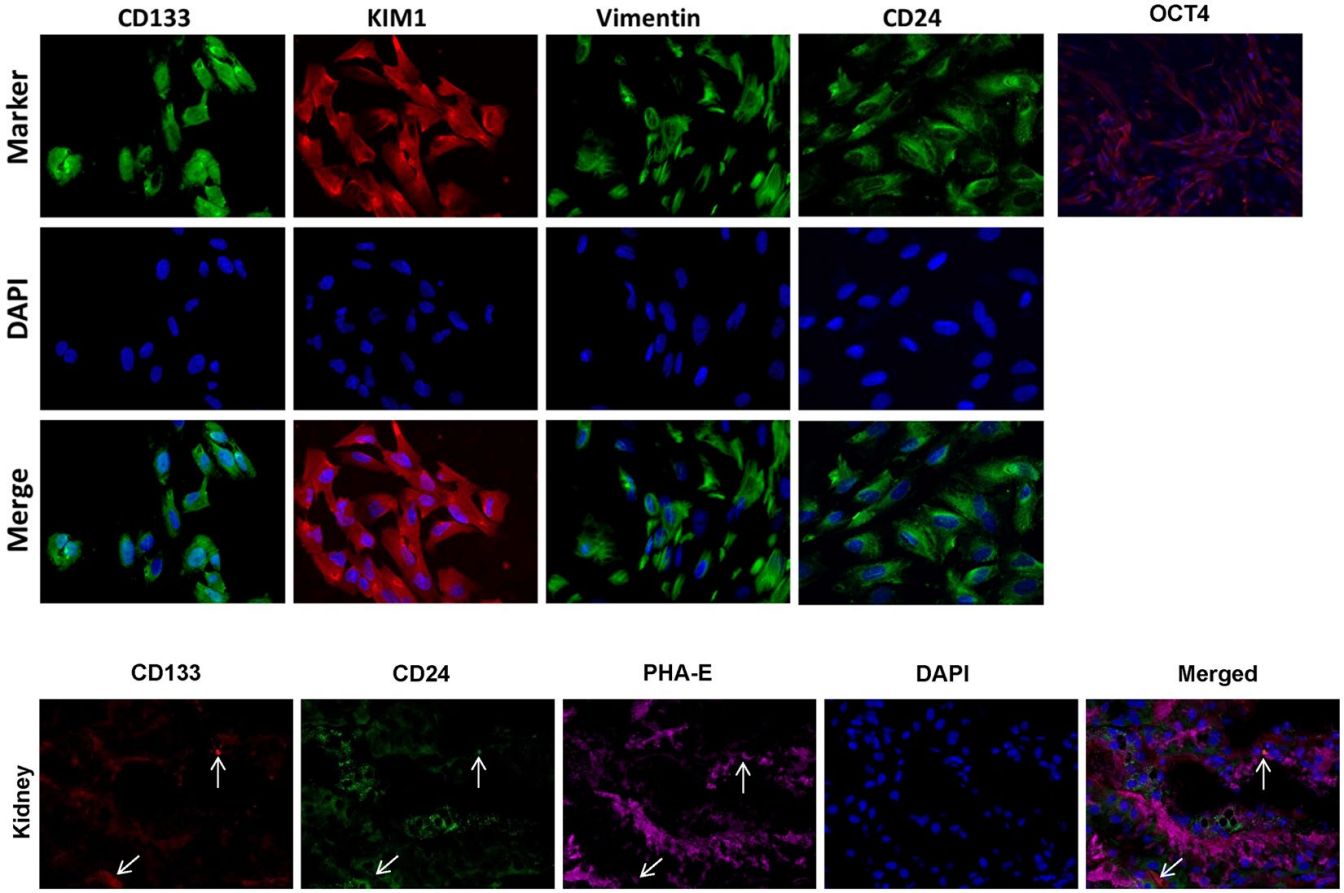
Characterization of scattered tubular cells (STC-like cells). Top: Immunocytochemistry staining confirmed that STC-like cells expressed CD133, KIM1, Vimentin, CD24, and OCT4. Bottom: Immunohistological staining of frozen pig kidney sections showed the presence of CD133^+^/CD24^+^ cells in proximal tubules *in-situ*.

**Figure 2:**
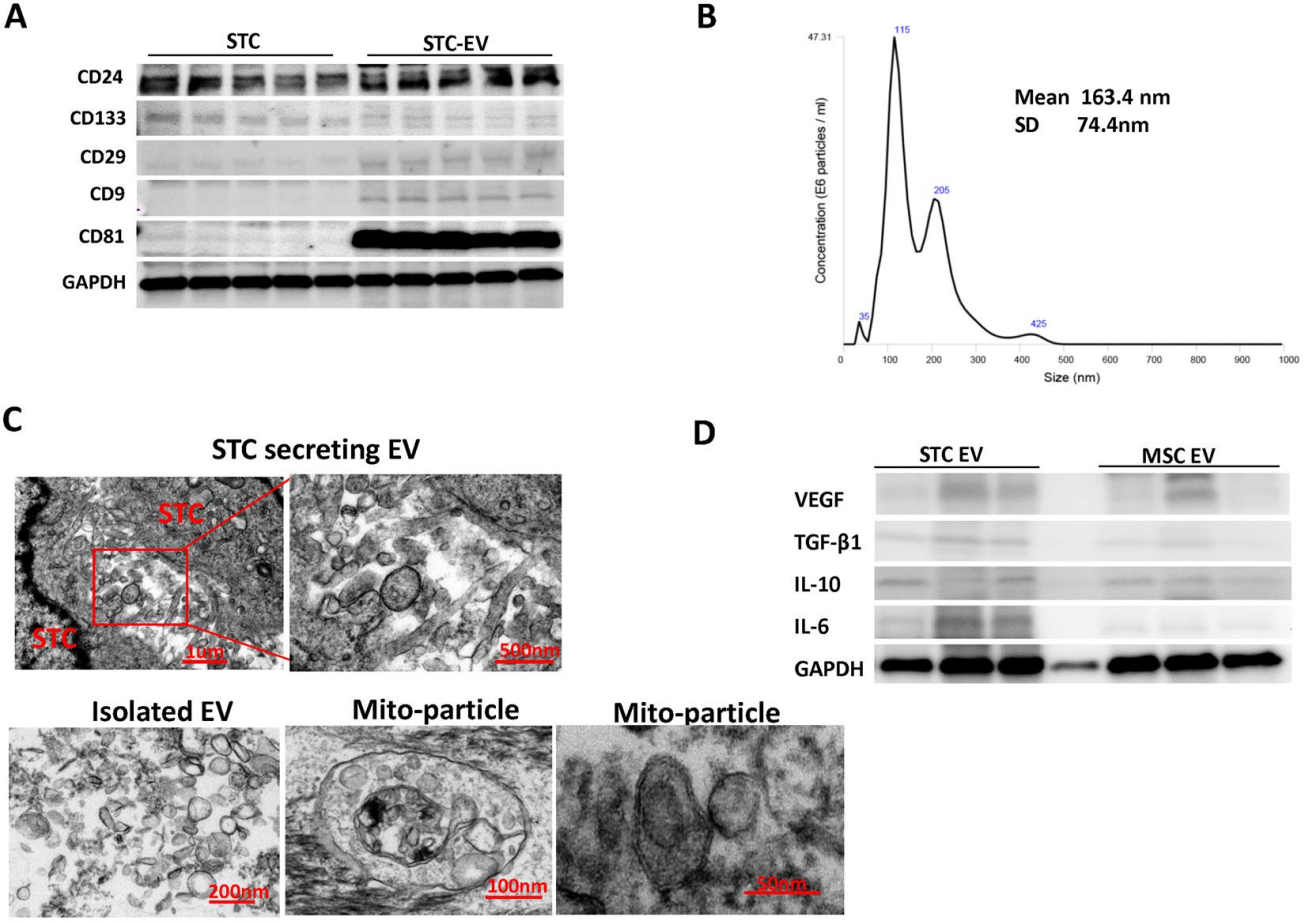
Characterization of scattered tubular cells (STC-like cells) derived extracellular vesicles (EV). (**A**) Western Blot analysis showed that the EV were positive for CD24, CD133, CD9, CD29 and CD81. (**B**) Nano-Sight analysis of purified EV to assess the size distribution of EV. (**C**)Transmission electron microscopy of cultured STC-like cells showed EV shedding from the cell surface, and in purified EV confirmed their spheroid shape and the presence of mitochondria packed-particles. (**D**) Western Blot analysis showed that STC-like cells EV express similar proteins to MSC EV, but more TGF-β1 and IL-6.

For characterization, primary STC-like cells were isolated from pig kidneys and analyzed for protein expression by immunofluorescence. STC-like cells expressed CD24, CD133, KIM1, Vimentin, and OCT4. In pig kidney sections, few CD133^+^/CD24^+^ cells were observed, particularly in the proximal tubules, supporting their renal presence *in vivo* (Fig. 1). STC-like cells-generated EV were isolated from the conditioned medium (CM) of STC-like cells, and Western Blotting showed them to be positive for CD133 and CD24, as well as for the typical EV markers CD29, CD9, and CD81 (Fig. 2A). EV ranged in size mainly from 100–300 nm as assessed by Nanosight analysis (Fig. 2B), and 10^8^ STC-like cells were found to release approximately 30 ug (2.6 × 10^12^) EV within 48 hours of serum starvation. Transmission electron microscopy demonstrated the release of EV from STC-like cells, and that purified EV show a homogenous pattern of spheroid particles. Isolated mitochondria-containing EV were also observed (Fig. 2C). Furthermore, STC-like cells-derived EV were compared to EV harvested from porcine adipose tissue-derived mesenchymal stem cells (MSC)^15^. Functional analysis showed that STC-like cells EV carried proteins that regulate angiogenesis (vascular endothelial growth factor, VEGF), inflammation (interleukin (IL)-6, IL-10), and fibrosis (transforming growth factor, TGF-β1) pathways, of which TGF-β1 and IL-6 were slightly higher than in MSC EVs (Fig. 2D).

### Protective effects of STC-like cells on Antimycin A (AMA)-induced cell injury via EV

**Figure 3:**
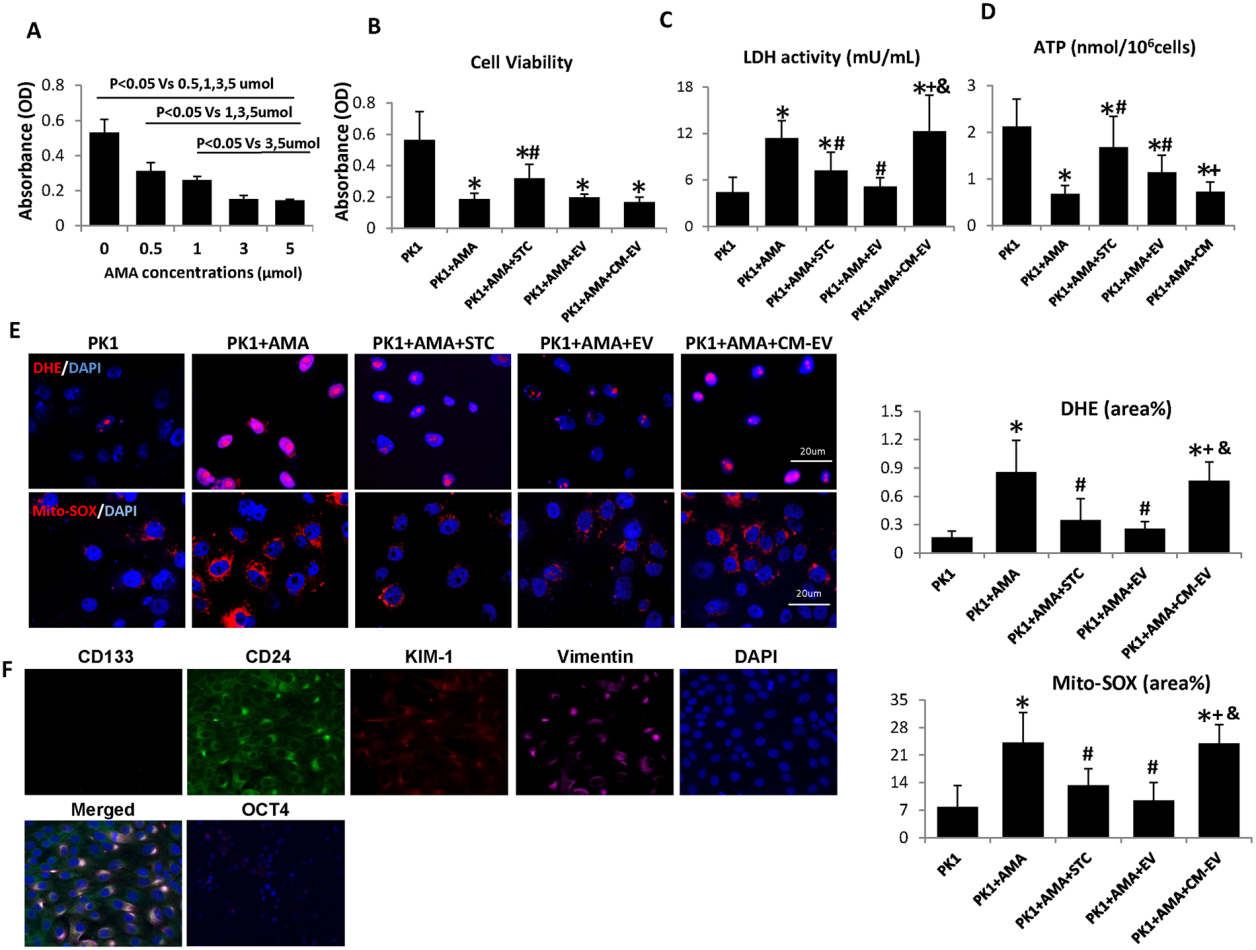
Scattered tubular cells (STC-like cells) and extracellular vesicles (EV) affect cellular injury. (**A**) Viability assays on PK1 tubular epithelial cells (TEC) showed that Antimycin A (AMA) reduced cell viability dose-dependently. (**B**) Cellular viability improved by co-cultured with STC-like cells, but not with EV or EV-free condition medium (CM-EV). (**C**) AMA increased Lactate dehydrogenase (LDH) activity, which decreased in STC-like cells and EV, but not CM-EV. (**D**) Compared to AMA alone, co-culture with STC-like cells and STC-like cells-EV partly restored energy production, but not CM-EV. (**E**) AMA significantly augmented DHE staining in TEC, which was reversed by STC-like cells and STC-like cells-EV, but not CM-EV. Mito-SOX showed a similar pattern in mitochondrial oxidative stress (*P < 0.05 vs PK1, ^#^P < 0.05 vs PK1 + AMA, +P < 0.05 vs PK1 + STC-like cells, & < P < 0.05 vs PK1 + AMA + EV). (**F**) Immunocytochemistry staining showed that PK1 cells expressed, CD24, KIM1, and Vimentin, but not CD133 or OCT4.

Firstly, dose-escalating assays were performed to select the appropriate dose of AMA, a mitochondrial ETC inhibitor mimicking ischemic injury, needed to decrease the viability of pig TEC (PK1 cells), as determined by the MTT assay. As shown in Fig. 3A, AMA reduced TEC viability in a dose-dependent manner and significantly inhibited TEC proliferation at 0.5,1,3 and 5 umol concentrations. 1 umol AMA was used for subsequent experiments, as it decreased viability by about 50%. When co-cultured with STC-like cells, the viability of pre-injured TEC was partly improved, whereas STC-like cells-EV and EV-free CM (CM-EV) had no effect (Fig. 3B). Lactate dehydrogenase (LDH) activity increased in the medium after treatment with AMA, but decreased significantly in TEC incubated with either STC-like cells or EV, while CM-EV lost this protective effect (Fig. 3C). AMA also attenuated cellular ATP production, which was improved by co-culture with STC-like cells or STC-like cells-EV, but not with CM-EV (Fig. 3D). Therefore, EV mimicked most of the protective benefits of STC-like cells on injured TEC *in vitro*.

### STC-like cells attenuate oxidative stress in AMA-treated TEC

Cellular and mitochondrial-derived oxidative stress was assessed by dihydroethidium (DHE) and Mito-SOX staining, respectively. AMA significantly augmented both DHE and Mito-SOX staining in TEC compared to untreated cells (Fig. 3E), an effect that was reversed by STC-like cells and STC-like cells-EV, but not CM-EV.

Importantly, although PK1 cells shared some features with STC-like cells, such as expression of CD24, KIM1, and Vimentin, but lacked CD133 and OCT4 expressions (Fig. 3F).

### STC-like cells transfer EV to TEC

**Figure 4:**
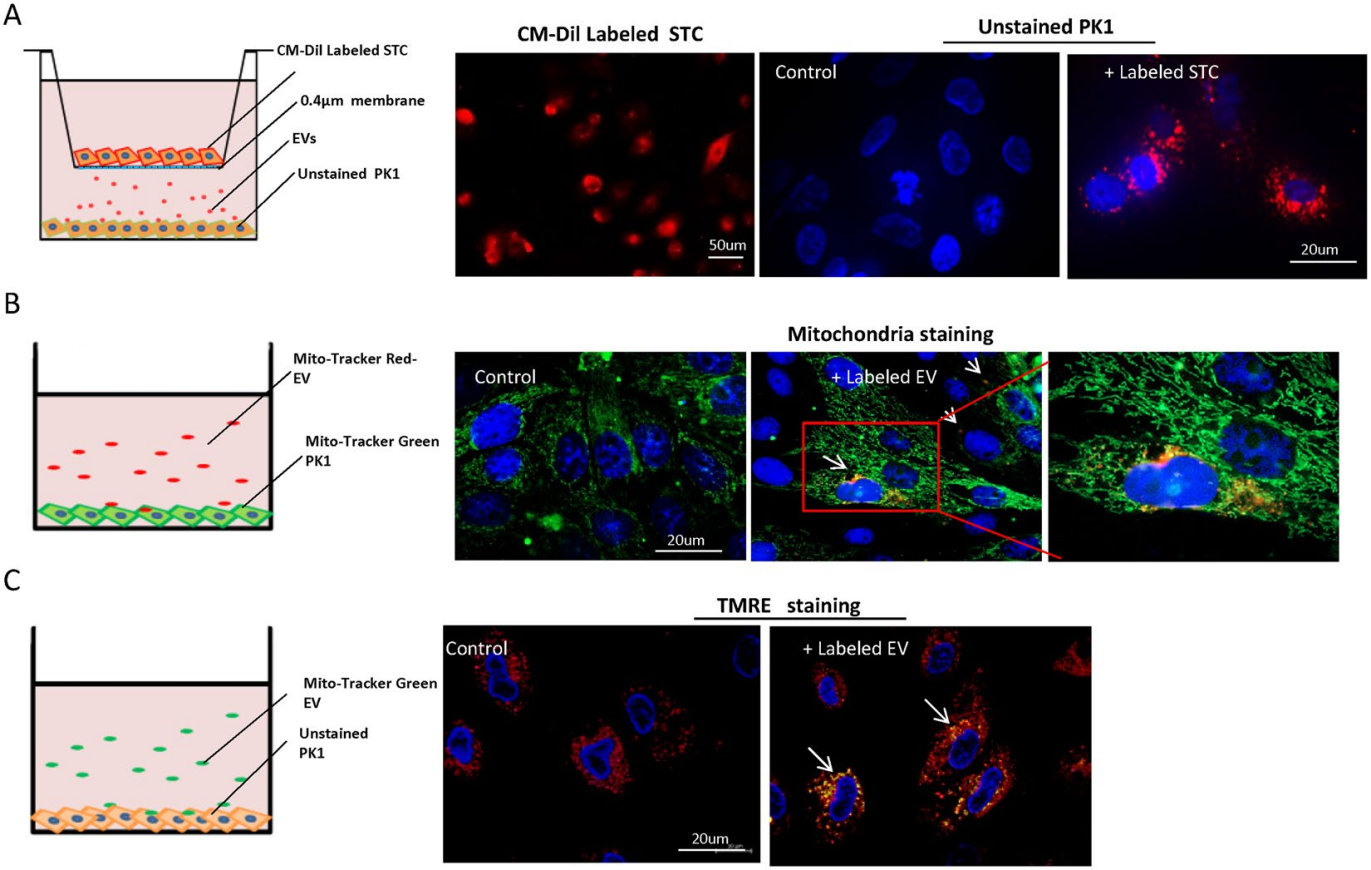
Delivery of mitochondria to tubular epithelial cells (TEC) through extracellular vesicles (EV). (**A**) STC-like cells labeled with red cellular membrane dye were co-cultured with un-labeled PK1 in a non-contact setting. Red signals were subsequently observed in unlabeled PK1 24 h after incubation, and unlabeled STC-like cells were used as control. (**B**) Isolated EV were labeled with Mito-Tracker red. After co-culture with Mito-Tracker green labeled TEC for 24 h, red and yellow staining was found in un-labeled PK1(white arrow). Unlabeled EV were used as control. (**C**) Mitotracker green labeled EV were incubated with PK1 for 24 hours. TMRE staining showed that transferred mitochondria were also stained (white arrow), suggesting maintained activity in injured PK1.

To explore whether STC-like cells transfer EV and mitochondria to TEC, STC-like cells were labeled with a red membrane dye and co-cultured with un-labeled TEC in a cell culture insert plate that prevents cellular contact, with unlabeled STC-like cells used as controls (Fig. 4A). Fluorescent microscopy identified red-labeled particles incorporated in TEC after 24 h of co-incubation with labeled STC-like cells, possibly due to transfer of labeled EV. In another experiment, isolated EV were pre-labeled with Mito-Tracker red or control to track their mitochondria (Fig. 4B). After co-culture with Mito-Tracker green pre-labeled TEC for 24 h, red and yellow fluorescence was found in the TEC, suggesting that STC-like cells-derived mitochondria were delivered to TEC and co-localized with host mitochondria. To test for the viability of donor mitochondria, mitochondrial membrane potential red staining was studied after incubation with Mito-Tracker green pre-labeled EV or control in TEC, which showed that transferred mitochondria maintained their membrane potential in injured TEC (Fig. 4C).

### EV regulate mitochondrial pathways in injured TEC

**Figure 5:**
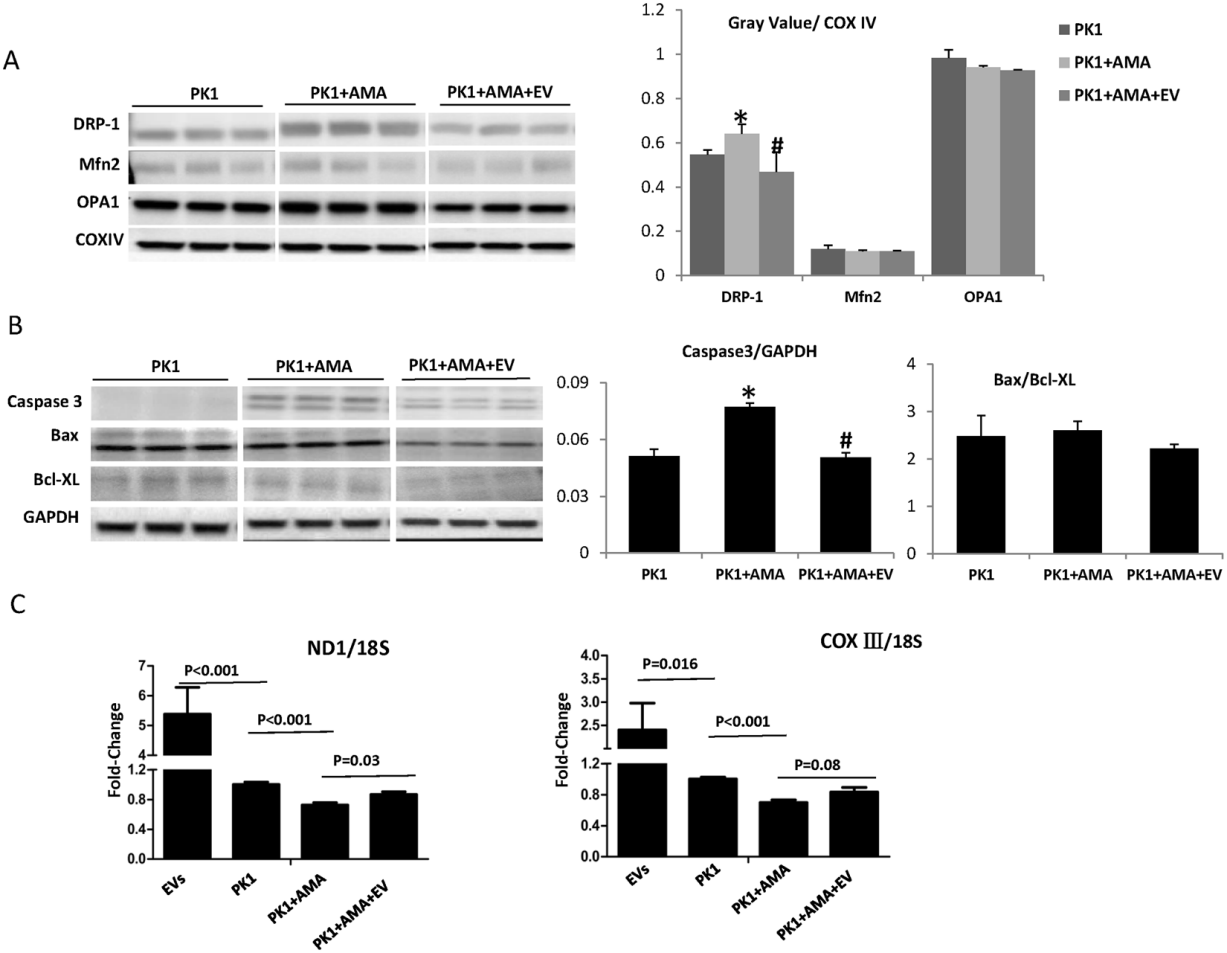
Mitochondrial regulatory proteins and mitochondrial DNAs. (**A**) After Antimycin A (AMA) exposure, the expression of dynamin-related protein1 (DRP1) in PK1 mitochondria increased significantly, but was attenuated by STC-like cells-EV. No significant changes were observed in fusion-related proteins Mitofusin 2 (Mfn2) and Optic Atrophy-1 (OPA1). (**B**) Pro-apoptosis caspase3 evaluated after AMA treatment in PK1, and EV down-regulated its expression. There was no change of Bax/Bcl-XL ratio. (**C**) ND1 and COXIII DNAs decreased significantly after AMA treatment in PK1. STC-like cells-EV contained mtDNAs and improved its expression in injured PK1 (*P < 0.05 vs PK1, ^#^P < 0.05 vs PK1 + AMA). The representative Western blots were cropped from the different parts of the same gel, and the full-length blots can be found in Supplementary info file.

In mitochondria isolated from TEC, AMA exposure increased protein expression of Drp1, which was abrogated by co-incubation with EV (Fig. 5A). No significant alterations were observed in mitofusion (Mfn)-2 or optic atrophy (OPA)-1. Pro-apoptotic caspase3 expression increased after AMA treatment in TEC, and EV down-regulated it (Fig. 5B), with no change in Bax/Bcl-XL ratio. Both NADH-ubiquinone oxidoreductase chain1 (ND1) and Cytochrome-C Oxidase (COX)-III mitochondrial DNA (mtDNA) expression, determined by PCR, decreased significantly in TEC after AMA treatment, suggesting mitochondrial injury. However, EV, which contained mtDNAs, inhibited the fall of mtDNAs in injured TEC (Fig. 5C).

### The effect of EV on murine renal hemodynamics and function

**Figure 6:**
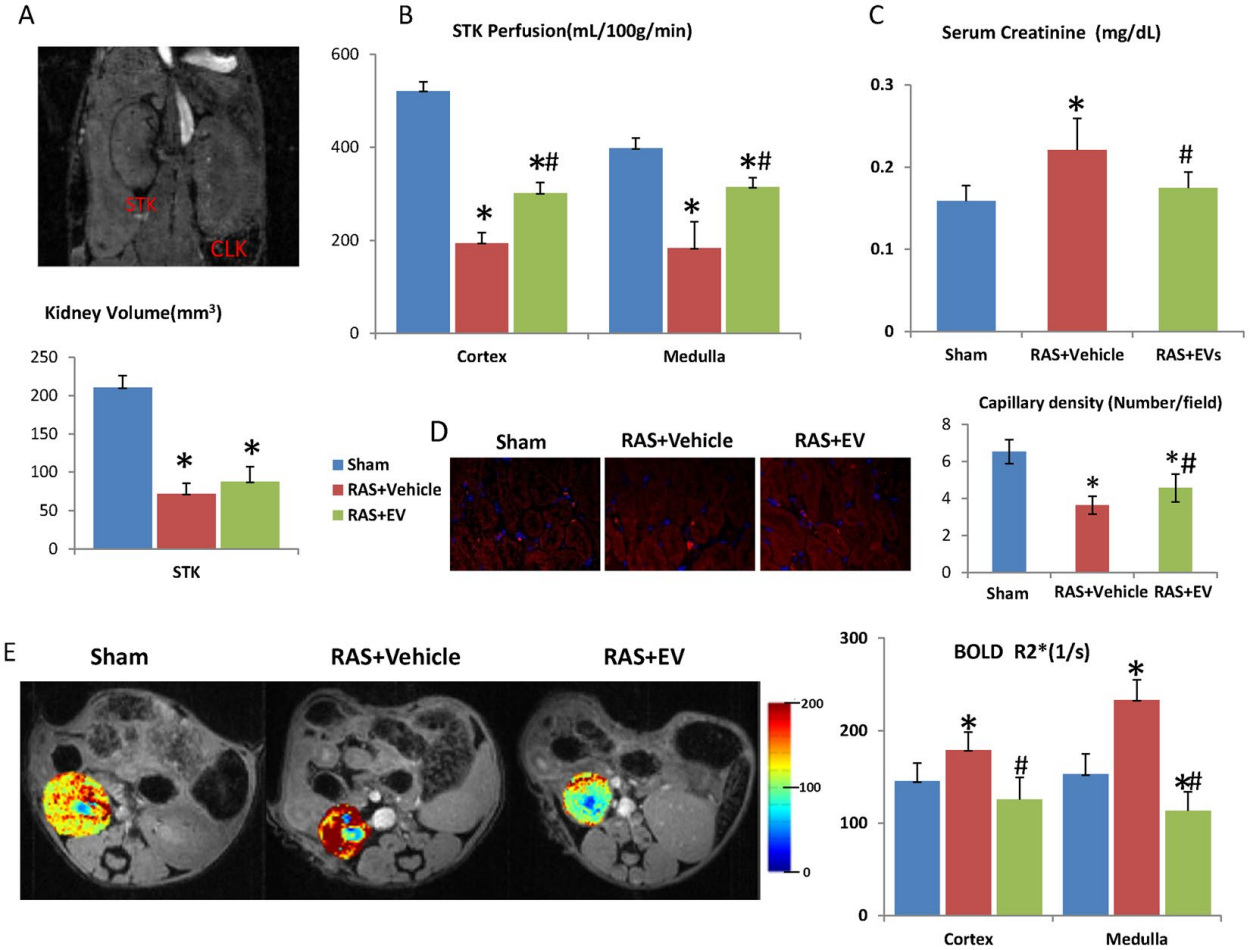
Renal hemodynamics in stenotic kidney (STK) of mice. (**A**) STK volume decreased significantly 4 weeks after RAS compared to Sham, and no significant difference from RAS + EV. (**B**) STK cortical and medullar perfusion showed a significant decrease, which was improved by EV. (**C**) Higher serum creatinine in RAS compared to the Sham 4 weeks after surgery, which was restored by EV. (**D**) Peritubular capillary density was significantly decreased in RAS + Vehicle, but improved after EV treatment. (**E**) Decrease of STK oxygenation was observed in RAS + Vehicle compared to the Sham both in the cortex and medulla, which was restored by EV (*P < 0.05 vs SHAM, ^#^P < 0.05 vs RAS + Vehicle).

STC-like cells-derived EV were injected into the carotid artery of RAS mice 2 weeks after surgery. Stenotic kidney (STK) volume, as measured by MRI, decreased significantly 4 weeks after surgery in RAS + Vehicle compared to Sham (Fig. 6A), with no significant effect of EV. On the other hand, STK cortical and medullary perfusion, which showed a significant decrease, was improved by EV (Fig. 6B). Serum creatinine increased in RAS + Vehicle compared to the Sham 4 weeks after surgery, and was normalized in RAS + EV (Fig. 6C). Furthermore, peritubular capillary density was significantly decreased in RAS + Vehicle, but improved after EV treatment (Fig. 6D). Similarly, kidney hypoxia assessed by blood oxygen level–dependent (BOLD) MRI was elevated in the RAS + Vehicle STK cortex and medulla, and EV restored STK oxygenation (Fig. 6E).

### STC-like cells-EV regulate mitochondrial pathways in the STK

**Figure 7:**
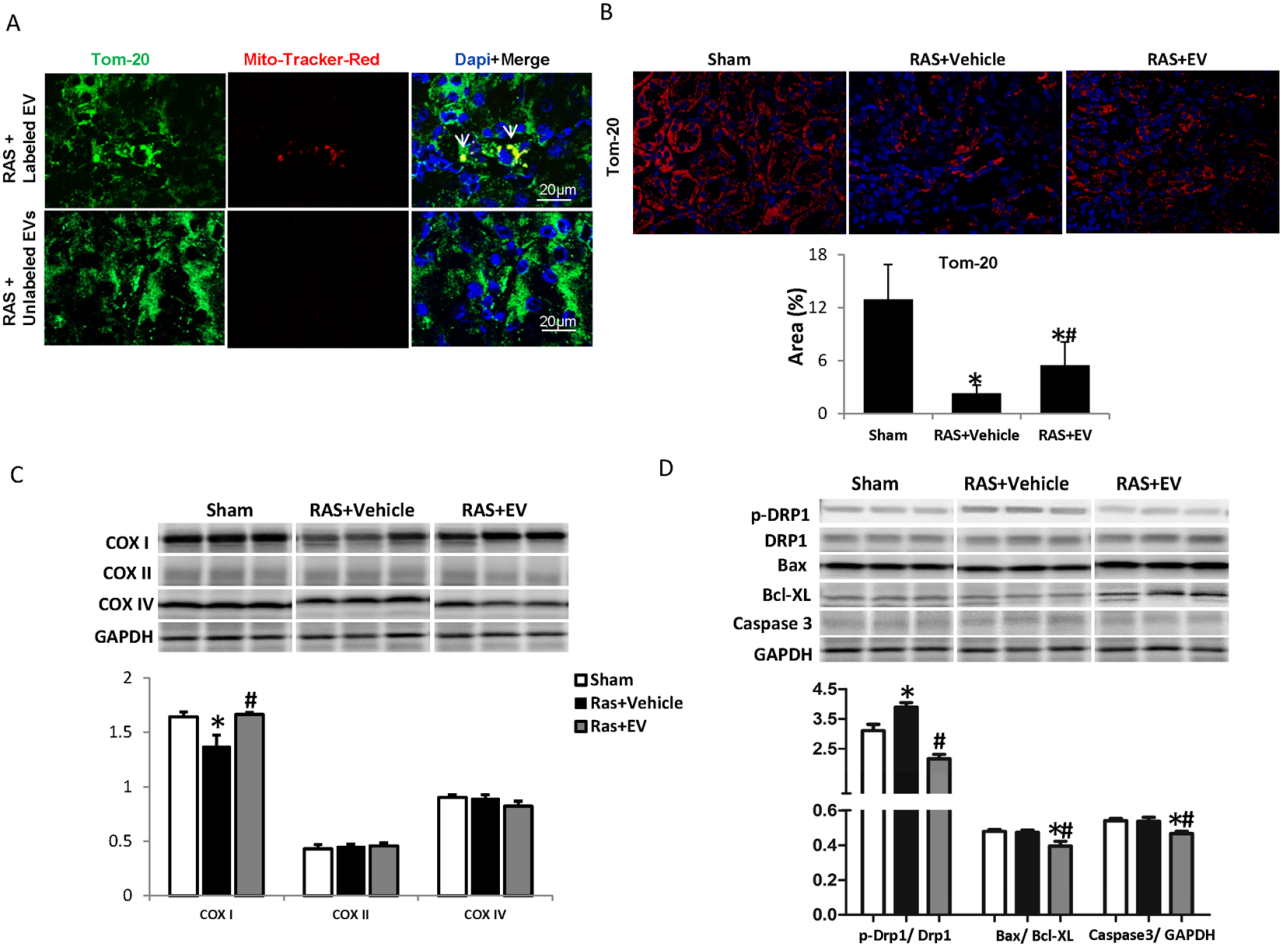
Mitochondrial regulation in the stenotic kidney (STK). (**A**) Representative images of Mito-Tracker-Red labeled EV (White arrows) in STK 2 weeks after delivery. Green shows endogenous mitochondria staining. (**B**) Tom-20 staining was used to assess renal mitochondrial density, which decreased in RAS STK and improved by EV. (**C**) RAS mice STK showed decrease of COXI expression, which was reversed by EV. No significant alterations were observed in COXII and COXIV. (**D**) The ratio of phosphorylated-Drp1/total Drp1 increased in STK, which was reversed by EV. There were no significant changes in caspase3, Bax/Bcl-XL ratio in STK, however, EV down-regulated both caspase3 and Bax/Bcl-XL ratio (*P < 0.05 vs SHAM, ^#^P < 0.05 vs RAS + Vehicle). The representative Western blots were cropped from the different parts of the same gel, and the full-length blots can be found in Supplementary info file.

24 h after injection of Mito-tracker-labeled EV, DiI-lableled EV or unlabeled EV into the carotid artery of mice with a 2-week RAS, EV and labeled mitochondria were found in the mouse STK (Fig. 7A). By two weeks after injection, Tom-20 staining, reflecting renal mitochondrial density, showed a decrease in the STK, which was improved by EV (Fig. 7B). The STK also showed a decrease in COXI expression, which was reversed by EV, but no significant alterations in COXII and COXIV (Fig. 7C).The ratio of phosphorylated/total Drp1 increased in the STK, but was reversed by EV(Fig. 7D). There were no significant changes in apoptosis-related caspase3 or Bax/Bcl-XL ratios in the STK, yet EV down-regulated both compared to sham.

### EV attenuated STK fibrosis

**Figure 8:**
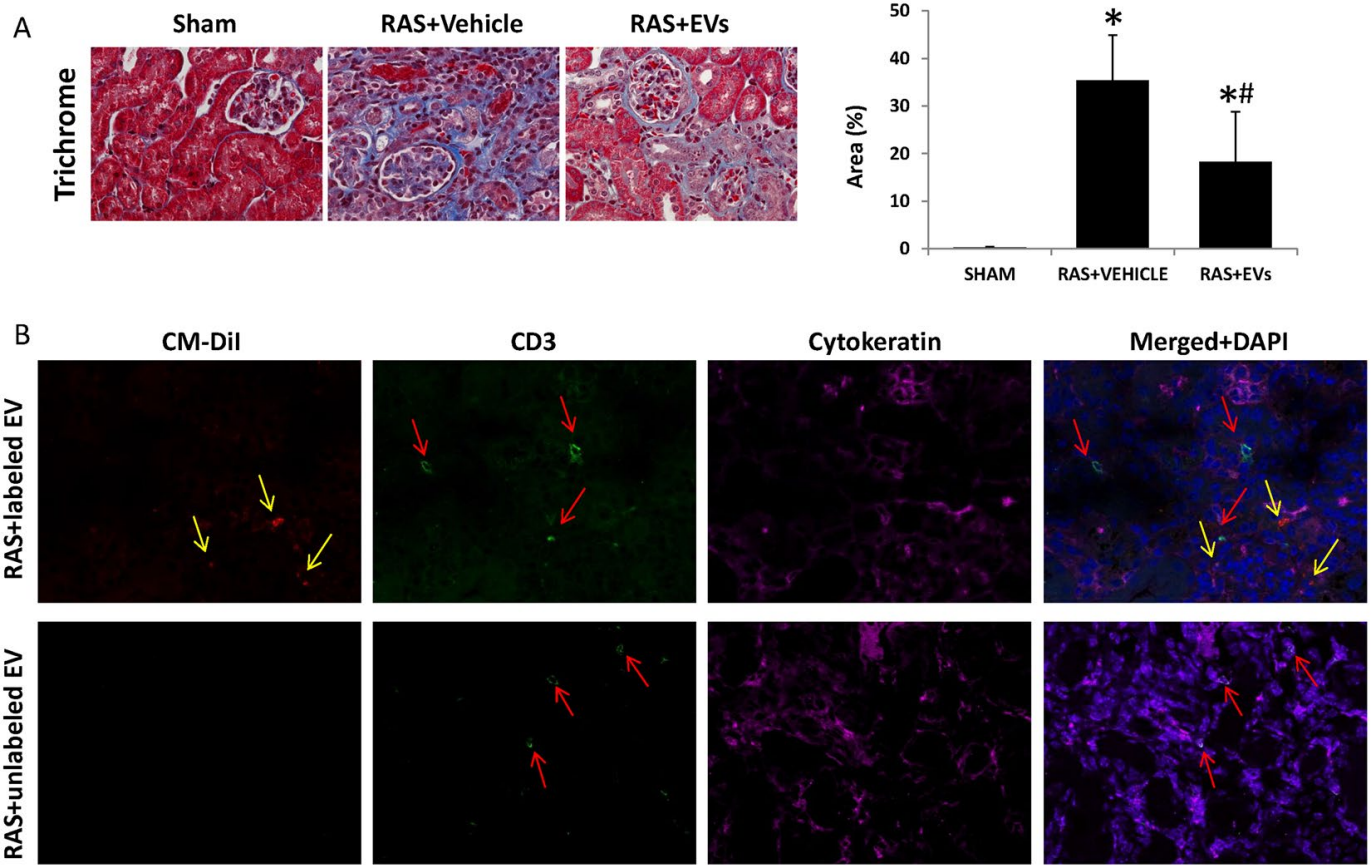
EV alleviated stenotic kidney fibrosis and improve its function. (**A**) Renal fibrosis increased significantly in RAS + Vehicle, whereas EV attenuated renal fibrosis. (**B**) Representative images of CM-Dil-labeled EV (red) and CD3-positive T cells (green) in the STK 2wks after delivery. Magenta shows tubular cell staining (cytokeratin). (*P < 0.05 vs SHAM, ^#^P < 0.05 vs RAS + Vehicle).

Four weeks after RAS, the STK showed marked interstitial fibrosis detected by Masson’s trichrome staining, which EV mitigated compared to RAS + Vehicle (Fig. 8A). Furthermore, the EV were found in STK 2 weeks after injection, and there was no obvious signs of immune response assessed by CD3 + T cells infiltration (Fig. 8B).

## Discussion

The current study demonstrates that primary-cultured STC-like cells exert a protective effect in AMA- injured TEC *in vitro* by releasing EV that are uptaken by TEC. Some of the EV may shuttle mitochondria or mitochondrial fragments to injured TEC, which may be recycled to restore mitochondrial functionality in the recipient cell. Furthermore, exogenously delivered mitochondria-containing EV may be integrated in the ischemic kidney tubules after systemic delivery, to improve mitochondrial pathways and alleviate chronic kidney injury *in vivo*. Hence, STC-like cells may communicate with injured TEC by releasing EV, which confer potent therapeutic benefits in ischemic kidney injury.

Dedifferentiation has been proposed as a central mechanism for proximal tubular repair. After injury, when many TEC are lost through apoptosis and necrosis, surviving TEC dedifferentiate to reconstitute the tubular epithelium^16^. However, recent studies demonstrated the existence of a rare resident population of TEC expressing putative stem cell markers CD24 and CD133, as well as other genes characteristic of proximal tubule dedifferentiation, such as vimentin and KIM-1. Termed STC, these cells expand in response to injury and their progeny reconstitutes the tubule^6^. Our studies support the existence of few resident CD133^+^/CD24^+^ cells in the pig kidney proximal tubules. However, given the relative scarcity of true STC *in vivo*, we cannot rule out the possibility that apart from bona fide STCs, the cells that we subsequently isolated included tubular cells undergoing dedifferentiation *in vitro* to express STC-like markers. Isolated STC-like cells were also positive for tubular cell specific injury marker KIM1, indicating that they originated from tubular cells. The paracrine mechanisms by which STC-like cells induce the repair process involves the release of EV, important vectors of inter-cellular communications and regulate the biological behavior of recipient cells. EV derived from microvesicular bodies or cell membrane budding often maintain some characteristics of their parent cells, which in our study included expression of CD24 and CD133, as well as the specific EV markers CD29, CD9 and CD81. Their size distributions suggest a mixed composition of exosomes and microvesicles, as we have previously shown^17^. Our study also suggested that proteins carried by STC-like cells EV may be involved in angiogenesis, inflammation, and fibrosis pathways, and were somewhat comparable to EV isolated from MSC, although they seem to express more TGF-β1 and IL-6.

Ischemia elicits kidney injury by decreasing oxygen delivery, impairing cellular oxygen utilization, and blunting energy production. AMA inhibits the ETC, hampers energy generation, and induces reactive oxygen species production and apoptosis, which mimic ischemic injury^18,19^. In our *in vitro* study, AMA attenuated cellular ATP production, and increased ROS production and cellular injury markers in cultured TEC. Co-culture with primary-isolated STC-like cells reversed the fall in ATP production and the increased LDH release, decreased ROS production, and slightly improved TEC viability. STC-like cells-derived EV manifested protective effects similar to STC-like cells, except for preservation of cell viability, implicating additional factors in this function. Interestingly, co-incubation with CM-EV abrogated most of the observed protective effects conferred by STC-like cells or EV on injured TEC. These observations suggest that much of the capacity on STC-like cells to decrease AMA-induced TEC injury resided in their EV.

Beneficial effects of tubular progenitor cells have been demonstrated in different kidney injury models, in which they induced erythropoietin production and promoted vascularization after acute renal tubular injury^20,21^. STC-like cells also acquire some progenitor cell characterizes and may confer similar salutary effects. This study shows that their beneficial effects may be partly mediated by release of EV, which are released by and possess somewhat similar characteristics to their parent cells. Further, their low immunogenicity and tumorigenic properties make EV attractive for allo-transplantation. Previous studies have demonstrated the safety and robust therapeutic effects of human cell-derived EV in rat injury models^22,23^. Here, swine STC-like cells-derived EV injected in RAS mice elicited no obvious signs of rejection. Moreover, STC-like cells-EV improved STK perfusion and restored its oxygenation in RAS mice, alleviated renal fibrosis, and improved renal function. This may also be partly attributable to the pro-angiogenesis effects of EV, as our previous study found MSC-derived EV contain angiogenesis-related miRNAs^17^. Indeed, in RAS kidneys peritubular capillary densities were improved after EV injection, possibly contributing to the improved perfusion.

Drp1, a key regulator of mitochondrial fission, contributes to cellular ROS production and apoptosis^24,25^ and its inhibition decreases caspase activity and apoptosis^26^. Previous studies have shown that ischemic injury upregulates Drp1 expression, and that its inhibition is protective in the ischemic heart and kidney^27–29^. Drp1 activity increased both in AMA treated TEC *in vitro* and in the chronically ischemic kidney, but was offset by STC-like cells-EV. STC-like cells-EV also reversed the elevation of apoptosis-related caspase3 and down-regulated Bax/Bcl-XL ratio in ischemic kidneys, consistent with anti-apoptotic properties. Persistent disruption of mitochondrial homeostasis may occur in ischemic kidney injury^30^. Both Mitochondrial DNAs in AMA-treated TEC and mitochondrial density (Tom-20) in the RAS-STK decreased significantly, but both were improved by STC-like cells-EV, implying that STC-like cells-EV may also augment mitochondrial biogenesis.

The transfer of various bioactive molecules contained in EV, including RNAs, proteins, and lipids, has been implicated in their tissue reparative power^31^. Furthermore, recent studies have shown that mitochondria can also be packaged in EV and transferred to target cells, constituting an additional mechanism by which they improve cellular energy^12,32^. Indeed, we detected mitochondria-containing particles in isolated STC-like cells-EV, which integrated within TEC in *vitro and* engrafted in the ischemic kidney *in vivo*. Remarkably, STC-like cells-derived and TEC mitochondria co-stained, suggesting co-localization and potential integration of the donor and recipient mitochondria. The EV mitochondria likely remained functional and acquired ETC function after their transfer to the injured TEC, as suggested by sustained membrane potential, which might have contributed to energy production *in vitro*. However, we cannot rule out the possibility that some of these viable mitochondria were of host origin.

Our study had several limitations. Firstly, cell-derived EV might ultimately change target cell phenotype and gene expression. Furthermore, EV tracking 2 weeks after injection was likely indirect, because pre-labeled EVs may have transferred the dye into recipient cells that interacted with them. Long-term follow-up studies are needed to evaluate long-term effects of STC-like cells-EV treatment in RAS mice. Moreover, in our study mitochondria-packaged EV integrated within injured TEC and conferred a protective both in *vitro* and in *vivo*. However, EV or their mitochondria cargo might eventually be consumed by macrophages^10^, so that the duration of their benefits needs to be determined. Lastly, given the spectrum of bioactive materials packed in EV^17^, the key functional determinants of renal protection warrant further experiments in the future. For example, the implication of higher IL-6 expression compared to MSC-EV needs to be clarified. Additional studies are also needed to fully characterize the cell population obtained under the experimental conditions described in this study.

## Conclusions

Our data show that STC-like cells exert a protective effect on injured tubular cells *in vitro*, and highlight EV as a key factor in this process, possibly partly via their mitochondria cargo. Moreover, STC-like cells-EV improved renal function and alleviated fibrosis in ischemic kidney injury in vivo. These findings shed a new light on mechanisms of kidney repair and may assist in development of new therapeutic strategies.

## Methods

### Animals

All protocols were approved by the Mayo Clinic Institutional Animal Care and Use Committee (protocol # A1609-16), all experiments were performed in accordance with relevant guidelines and regulations. STC-like cells acquired from domestic pig kidneys were used for the studies. Frozen kidney sections from 2 pigs (6 month old, normal diet) were used for double-staining of CD133 (1:100, Novus Biologicals) and CD24 (1:100, Abcam) to detect resident STC-like cells, and phaseolus vulgaris erythroagglutinin (PHA-E, Invitrogen) as a proximal tubular marker.

Male 129-S1 mice (Jackson Lab, ME, 11 weeks of age) were studied for 4 weeks, randomly divided into Sham (n = 8), RAS + Vehicle (n = 10), and RAS + STC-like cells-EV (n = 10) groups. RAS was induced by surgical placement of a 0.15 mm diameter arterial cuff, whereas sham surgeries without placement of a cuff were performed in the control group, as previously described^33^. After 2 weeks the carotid artery was cannulated via a vascular cut down, and 200 uL PBS or STC-like cells-EV (30 ug in 200 uL PBS) slowly injected caudally. In some mice, the STK was obtained from Mito-Tracker or CM-Dil labeled EV-treated mice for EV tracking 2 weeks after injection (n = 4 each group). All remaining mice were scanned with MRI, 2 weeks after injection, and subsequently euthanized with CO_2_. Kidneys and blood samples were collected for *ex-vivo* studies.

### Cell culture

For *in vitro* studies, pig proximal kidney TEC (LLC-PK1, ATCC, Manassas) were cultured in Medium-199 (Gibco BRL, USA) containing 3% FBS^15^. STC-like cells were isolated from fresh pig kidneys (6 month old, normal diet) as previously described^34^, with few modifications. Briefly, 3–5 g pig kidneys including both cortex and medulla were sectioned and washed with PBS. Kidneys were diced and digested with 2 mg/ml collagenase for 1 hour, then forced through a 60-mesh (250-μm) steel sieve to remove the fibrous component. The cellular fraction was then passed through 100 μm cell strainer and followed by the addition of Medium 199 containing 3% fetal bovine serum (Gibco BRL, USA) at 37 °C in a humidified atmosphere with 5% CO_2_. The culture medium was replaced every 2 days to remove non-adherent cells. After about two weeks, the adherent cells were harvested with 0.25% trypsin (Gibco BRL, USA) treatment and sub-cultured. Phenotypic analysis of cultured STC-like cells (as well as PK1 TEC) employed immunofluorescence for CD133 (Novus Biologicals), CD24 (Abcam), KIM1 (R&D Systems), Vimentin (Abcam), and OCT4 (Abcam).

### Isolation and surface marker analysis of EV

EV were obtained from supernatants of STC-like cells as previously described^17,35^. Briefly, STC-like cells were cultured in M199 medium without serum for 48 hours. The CM was centrifuged at 2000 g for 20 minutes to remove debris, and then ultracentrifuged at 100,000 g in a SW41 swing rotor (Beckman Coulter, CA) for 1 hour at 4 °C. The supernatant was collected and used as CM-EV. The EV were washed once with M199, and submitted to a second ultracentrifugation. Protein content and size of EV were assessed by the Bradford assay and Nanosight technology (Nanosight, London, UK), respectively. Western Blot was used to characterize the CD24 (Abcam), CD9 (Bio-Rad), CD81, CD133, (both AVIVA Systems Biology), and CD29 (AbD Serotec) markers. To evaluate proteins carried by EV, Western Blotting of VEGF, IL-6, IL-10, TGF-β1 were performed and compared to EV isolated from adipose tissue-derived MSC isolated from comparable healthy pigs^15^.

### Transmission electron microscopy

STC-like cells were fixed with 2.5% glutaraldehyde in PBS for 2 h and transmission electron microscopy (HITACHI, Japan) performed on slides embedded in epoxy resin. Ultrathin sections were stained with uranyl acetate and lead citrate. EV were fixed with 2.5% glutaraldehyde in PBS for 2 h, after washing; EV were ultra-centrifuged and suspended in 100 uL PBS. A 20 uL of EV was loaded onto a formvar/carbon-coated grid, negatively stained with 3% aqueous phosphor-tungstic acid for 1 minute, and then observed^35^.

### Assessment of cell viability

A quantitative colorimetric assay with 3-(4, 5-Dimethyl-2-thiazolyl)-2, 5-diphenyl-2H-tetrazolium Bromide (MTT) (Roche Diagnostics, Germany) was utilized to determine cell viability. After achieving an 80% confluence, the medium was replaced with different concentrations of AMA (0, 0.5, 1, 3, 5 umol) in PBS for 1 hour. After incubation, the medium was replaced for 72 h with serum-free culture medium (M199). In co-culture experiments TEC were treated with 1 umol AMA and incubated with serum-free culture medium, EV-free STC-like cells CM, serum-free medium with EVs (about 15 ug/mL), or serum-free medium with STC-like cells. At the end of each time point, the MTT assay was performed according to manufacturer’s instructions. Absorbance was measured at 570 nm.

### DHE and MitoSOX

DHE (Molecular Probes, Eugene, OR) staining was used to assess cellular ROS production. Cells were seeded on chamber slices and incubated with 10 μM DHE for 40 min, washed and viewed using fluorescence microscopy. To assess mitochondrial ROS production, MitoSOX (2 μM, Molecular Probes) was incubated with cells for 20 min. Nuclei were stained with Hoechst 33342 (Sigma Aldrich, USA). Intracellular fluorescence was then quantified using AxioVision (Carl Zeiss MicroImaging, NY) software. Eighteen high-power fields were selected randomly for each group for quantification^36^.

### LDH and ATP assay

LDH Assay Kit (Abcam, ab102526) was used to measure LDH activity in the culture medium following manufacturer’s instructions. Media were centrifuged at 2000 g for 20 minutes to remove cell debris, and collected for the subsequent steps. Cell ATP production was tested by the ATP Assay (Abcam, ab83355). 10^6^ cells were harvested and homogenized, centrifuged to remove insoluble material, and the supernatant collected for the following steps.

### Mitochondria isolation and Western Blot

Mitochondria were isolated from PK1 cells using a mitochondria isolation kit (Thermos Fisher, USA), following vendor’s instructions, as we have shown^37^. Protein concentration was measured with Bradford Protein Assay. Specific antibodies against Drp1 (1:1000; Abcam), Mfn2 (1:1000; Abcam) and OPA1 (1:500; Abcam) were used with blotting protocols. COXIV (1:5000; Abcam) was used as loading controls. Cell surface, EV or kidney protein expression was studied in homogenate. Specific antibodies against CD9 (1:5,000; Abcam), CD81 (1:1,000; Abcam), CD29(1:1000, Abcam), CD24(1:500, Abcam), CD133(1:500, Aviva Systems Biology), COX I (1:1000, Abcam), COX II (1:1000,Abcam), Caspase3 (1:1000, Abcam), Bax(1:1000,Abcam), Bcl-XL (1:1000, Abcam), COX IV, Drp1, p-Drp1(1:1000, Abcam) antibodies were used with blotting protocols, and GAPDH (1:1000, Abcam) as loading controls.The density of each band was analyzed by Image-Pro Plus 6.0 software.

### STC-like cells-EV uptake by TEC *in vitro*

We used the CM-Dil (Molecular Probes, USA) dye to label STC-like cells for 20 minutes, washed cells with PBS twice, and incubated with TEC in a 0.4 μm Trans-well (Sigma-Aldrich) for 24 h. This size allows transfer of fluids and small EV, but not STC-like cells^38^. Unlabeled STC-like cells were used as controls. Hoechst 33258 dye was added for nuclear staining and results observed under confocal microscopy.

### Mitochondria labeling in STC-like cells-EV and TEC

After isolation of EV from the CM, the MitoTracker® Red (Invitrogen) dye was added to suspension, EV were washed twice with M199, underwent ultracentrifugation as described. TEC were seeded in the chamber slides and stained with MitoTracker® Green (Invitrogen). After double washing of cells, MitoTracker® Red labeled EV (15 ug/mL) were added for 24 hours. Hoechst was added for nuclear staining.

### TMRE staining in cultured TEC

EV were stained with MitoTracker® Green FM (Invitrogen) as described above, and 15 ug/mL added to TEC for 24 hours. TMRE (Sigma-Aldrich) staining followed manufacturer’s instructions, and nuclei were stained with Hoechst.

### Quantitative real-time PCR

Total DNAs were isolated from EV or PK1, and the expression of target DNAs quantified using real-time PCR with Taqman chemistry (Applied Biosystems, Carlsbad, USA). 18 S DNA was used as an internal normalizer. Real-time PCR was carried out using the following primers: COX III, *F-*GGAGCCCTATCAGCCCTTTTAATA, *R-*TTGTCAAAGTATTGGTTAATAGTCCTAGAGATAGT. ND1, *F-*CAAGCCTAGCAGTCTACTCTATCCT, *R-*GATTGTTTGGGCTACTGCTCGTA. 18 S, *F-*GCCCGAAGCGTTTACTTTGAA, *R-*CATTATTCCTAGCTGCGGTATCCA.

### Imaging protocol and data analyses

Four weeks after RAS or Sham surgery, renal volume and hemodynamics were assessed, as we previously described^33^. Renal volume was quantified from images acquired using a respiration-gated three-dimensional fast imaging. Renal perfusion was measured with arterial spin labeling, and quantified from the flow-sensitive alternating inversion-recovery sequence, with rapid acquisition with relaxation enhancement images. Renal oxygenation was assessed with BOLD MRI. Eight images were reconstructed after zero-filling the k-space data to 256 × 256. T2* was quantified by pixelwise monoexponential fitting on the averaged magnitude of all eight images over echo times. R2* (1/T2*) was used as an index of blood oxygenation level.

### STC-like cells-mitochondria tracking and mitochondrial density analysis in STK

For tracking experiment, STK were acquired 2 weeks after Mito-Tracker or CM-Dil labeled EV-treated mice. In kidney sections, mitochondria were labeled with rabbit anti-mouse TOM-20 antibody (Abcam) followed with goat anti-rabbit second antibody. TOM-20 positive staining was quantified randomly under confocal microscopy in 10–15 fields in each section two weeks after EV treatment, as a surrogate for mitochondrial density. In CM-Dil labeled EV-treated mice, the proximity to CD3 (Abcam) positive T-cells was examined in kidney sections to evaluate possible immune rejection, and tubular cells were stained with cytokeratin (Abcam).

### Serum creatinine

The level of serum creatinine was determined using a commercial kit (DetectX® Serum Creatinine kits). Briefly, 25 μL of standards or samples were pipetted into a microliter plate, and the color generating reaction was initiated with the Creatinine Reagent. The concentration of creatinine was calculated using the delta of the optical density readings at 30 and 1 minute compared to the curve generated from the standards.

### Fibrosis scoring

Kidney fibrosis was tested by Masson Trichrome staining, assessed in 5-um sections of each kidney using AxioVision software. Fibrosis score (% area stained) was quantified randomly in 10–15 fields in each section^39^. Furthermore, peritubular capillary densities were manually quantified using kidney sections immunohistologically stained with CD31 (1:100, Biolegend), averaged from 10 fields.

### Statistical analysis

Statistical analysis utilized the JMP software, and data expressed as mean ± standard deviation. Statistical significance was assessed by one-way analysis of variance (ANOVA) followed by unpaired t-test for normally distributed data or non-parametric (Wilcoxon and Kruskal-Wallis) test for no-normally distributed data. A value of P ≤ 0.05 was considered to be statistically significant.

## Electronic supplementary material

Supplementary data
