# Supplementary material for: Renal scattered tubular-like cells confer protective effects in the stenotic murine kidney mediated by release of extracellular vesicles
Source: Sci Rep. 2018 Jan 19;8:1263. doi: 10.1038/s41598-018-19750-y (PMC5775303; doi:10.1038/s41598-018-19750-y)
Supplement: Supplementary file 1 — Supplementary data [file 41598_2018_19750_MOESM1_ESM.pdf]

Renal scattered tubular-like cells confer protective effects in the stenotic murine kidney mediated by release of extracellular vesicles

Xiangyu Zou[1,2] MD, PhD, Soon Hyo Kwon[1,3] MD, Kai Jiang[1] PhD, Christopher M. Ferguson[1] M Sci, Amrutesh S. Puranik[1] PhD, Xiangyang Zhu[1] MD, PhD, Lilach O. Lerman[1] MD, PhD

[1]Division of Nephrology and Hypertension, Mayo Clinic, Rochester, MN, USA
[2]Department of Urology, Xinhua Hospital, Shanghai Jiao Tong University School of Medicine, Shanghai, China
[3]Division of Nephrology, Soonchunhyang University Seoul Hospital, Korean

Original blots for Figure 5

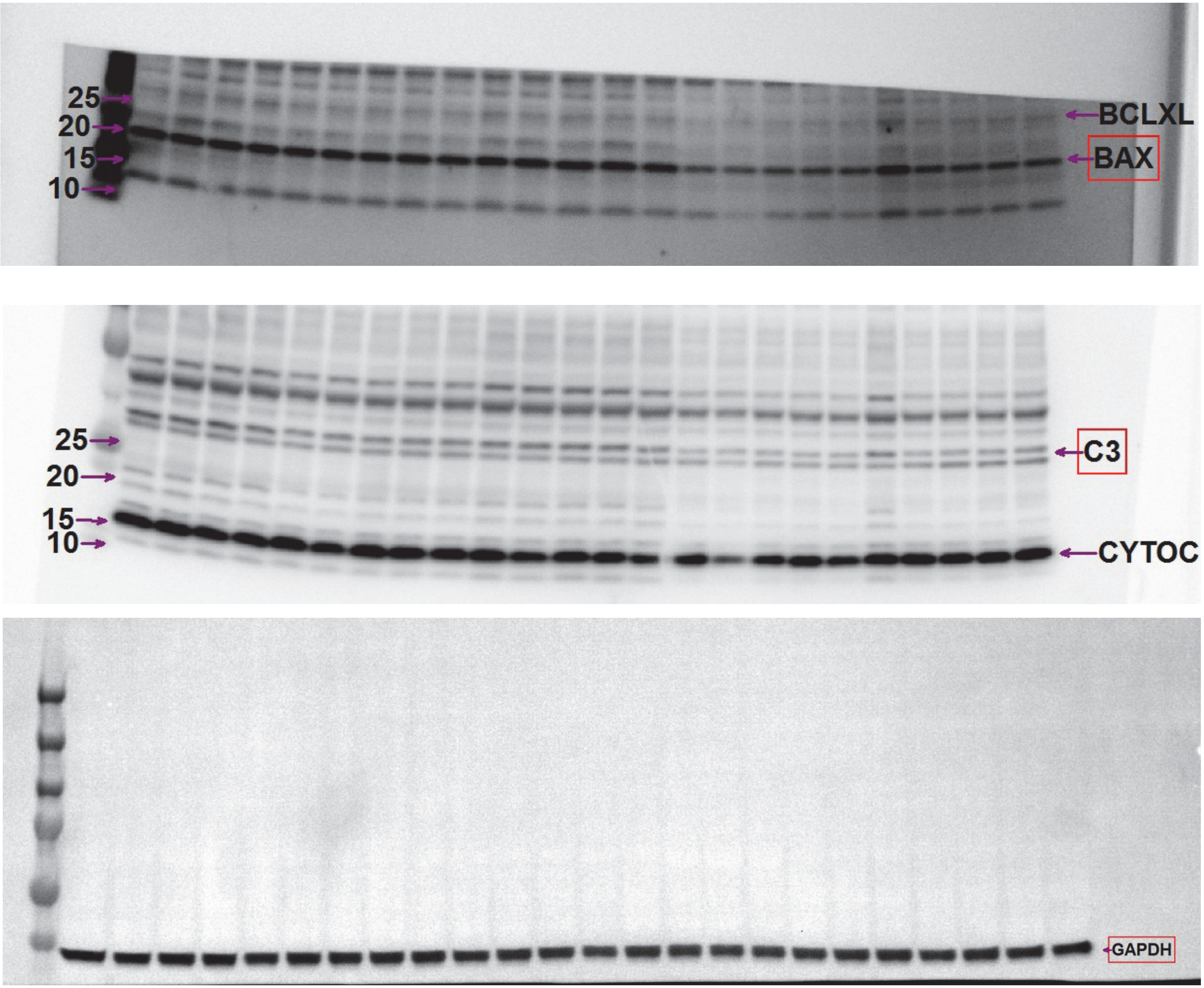

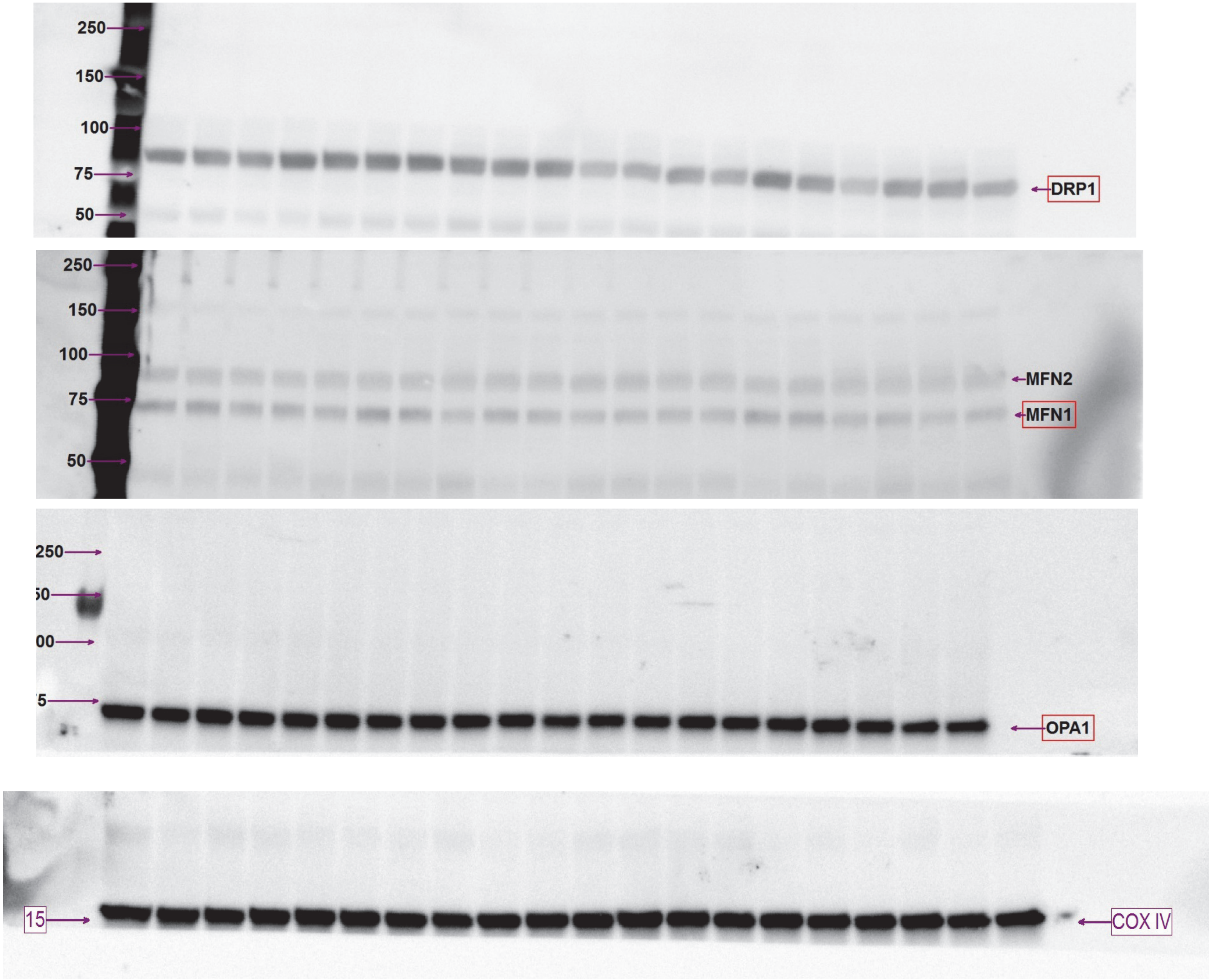

250
150
100
75
50
DRP1
250
150
100
75
50
MFN2
MFN1
250
50
00
5
OPA1
15
COX IV

Original blots for Figure 7

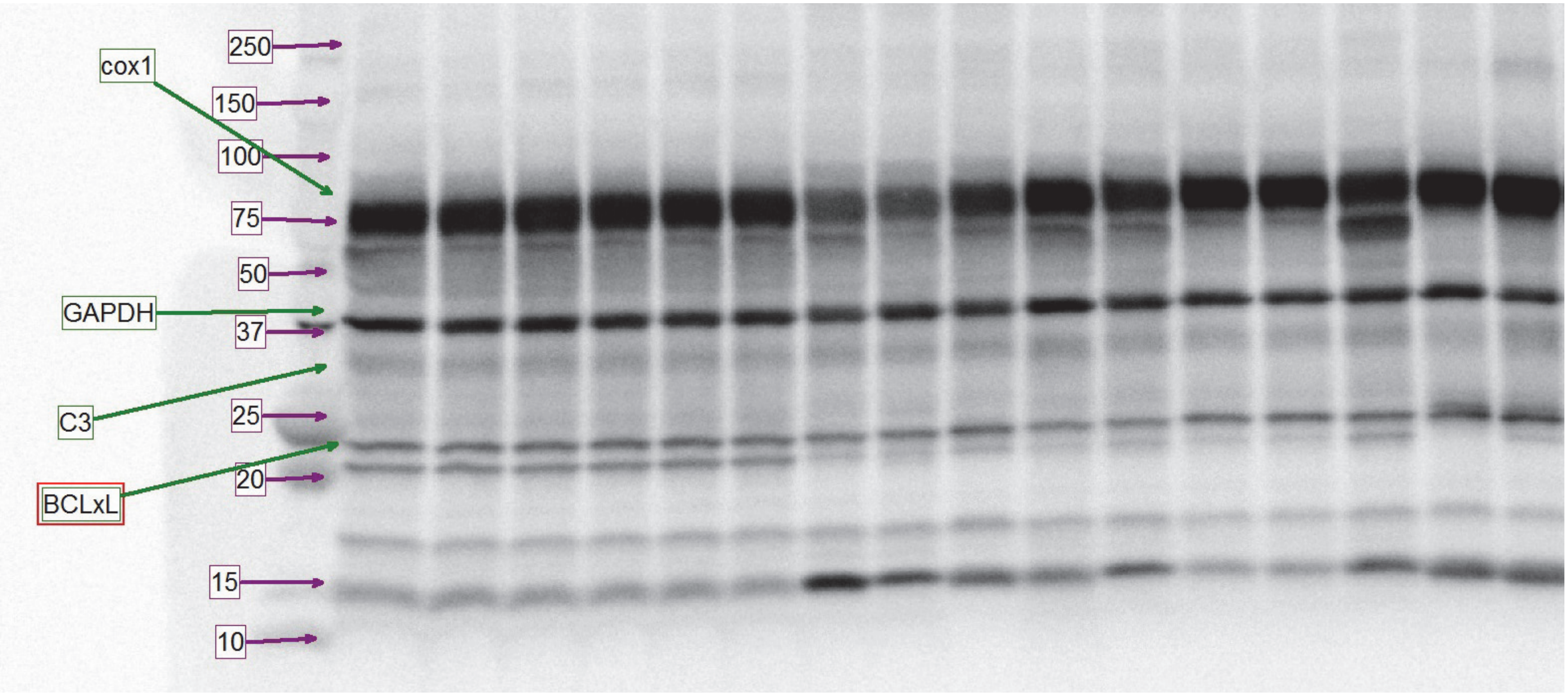

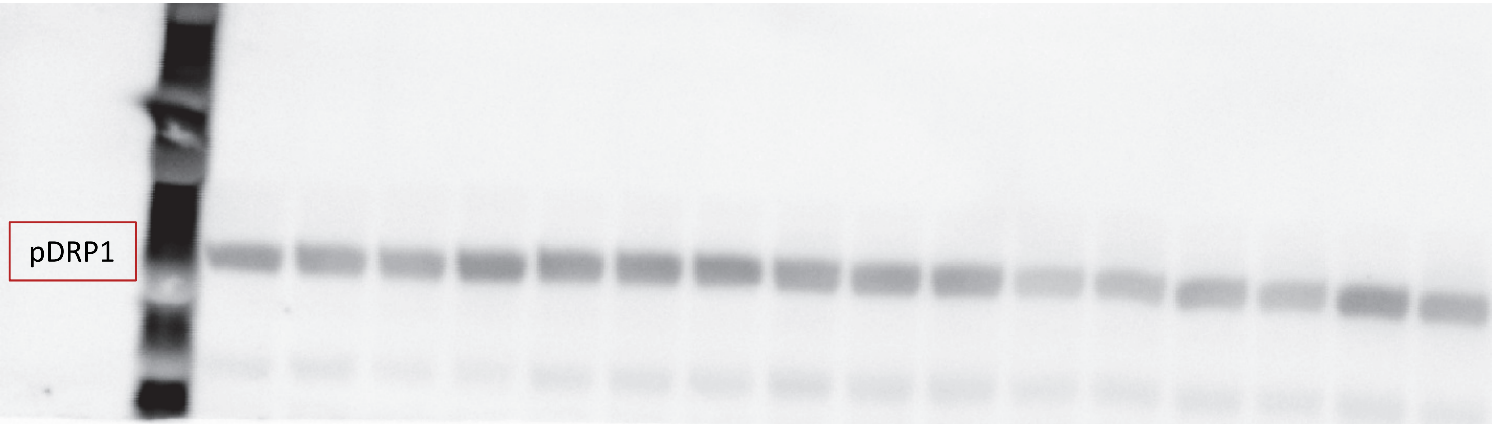

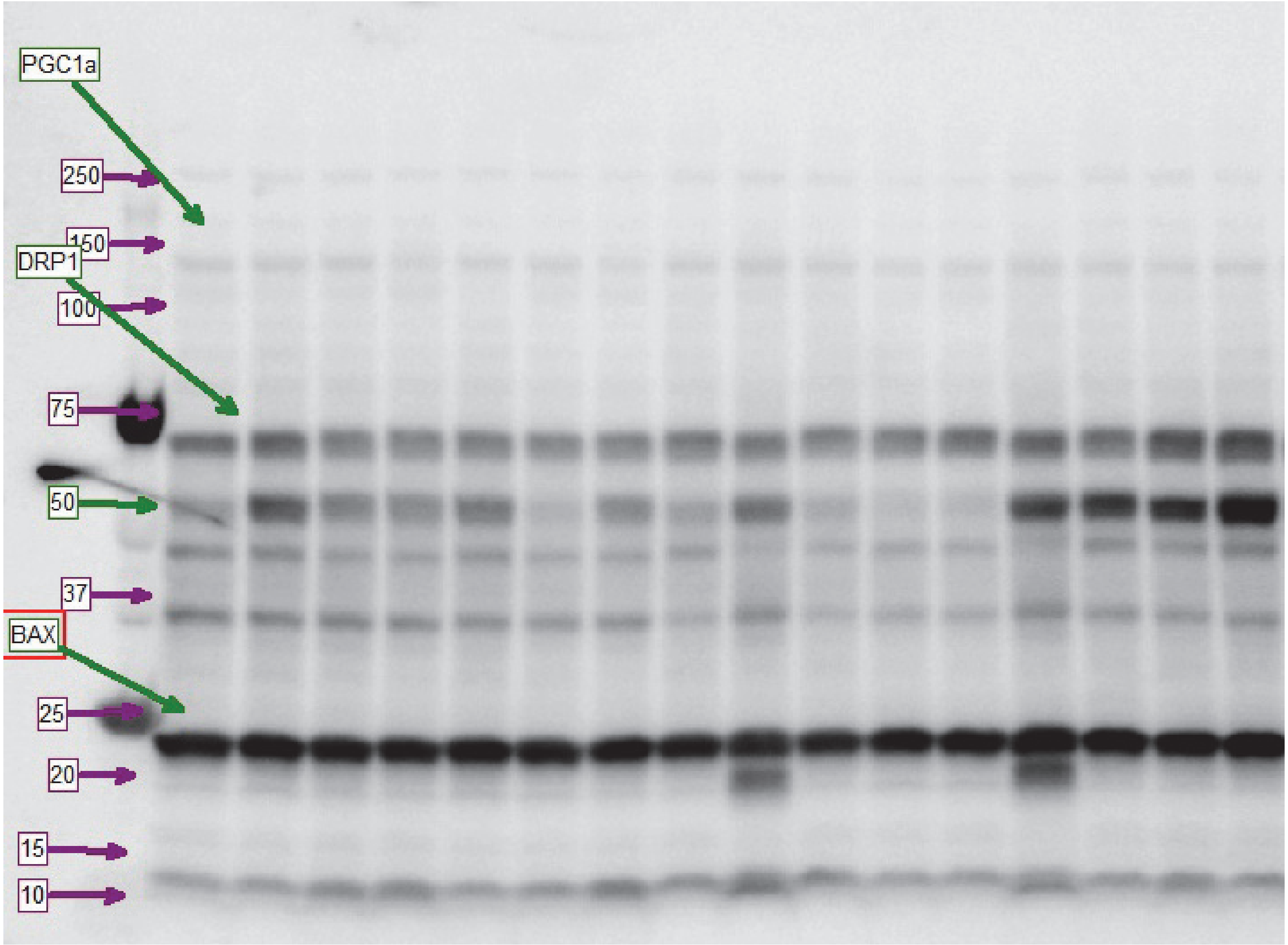
PGC1a
250
150
DRP1
100
75
50
37
BAX
25
20
15
10

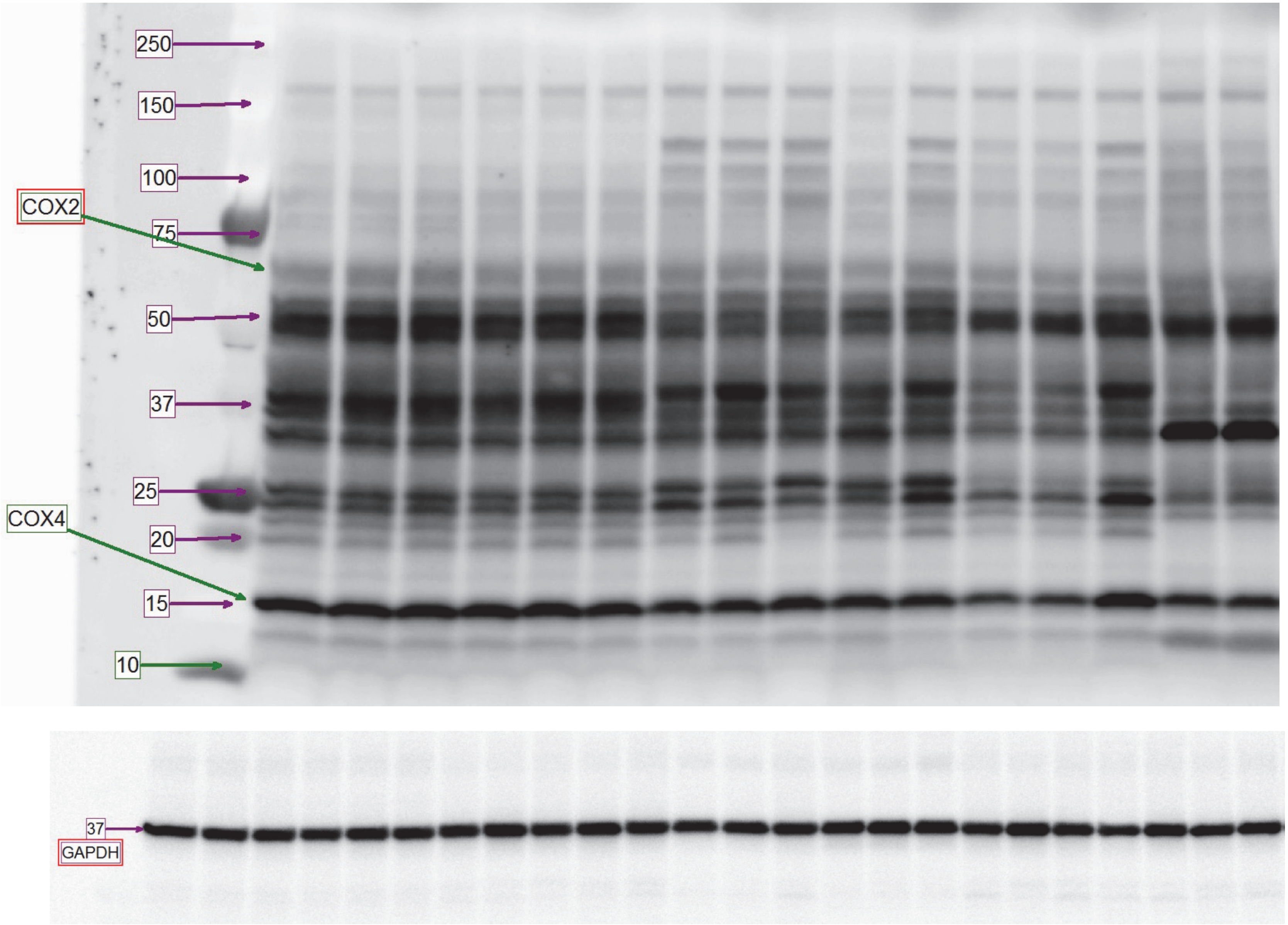
250
150
100
COX2
75
50
37
25
COX4
20
15
10
37
GAPDH
